# Role of calcium homeostasis in retinal ganglion cell degeneration

**DOI:** 10.4103/NRR.NRR-D-24-01651

**Published:** 2025-04-29

**Authors:** Sean McCracken, Philip R. Williams

**Affiliations:** John F. Hardesty, MD Department of Ophthalmology and Visual Sciences, Washington University School of Medicine, St. Louis, MO, USA; Department of Neuroscience, Washington University School of Medicine, St. Louis, MO, USA; Hope Center for Neurological Disorders, Washington University School of Medicine, St. Louis, MO, USA

Calcium (Ca^2+^) is a key intracellular messenger involved in a variety of cellular functions. Intracellular Ca^2+^ dysregulation drives neuron cell death in multiple degenerative diseases and traumatic conditions. Retinal ganglion cell (RGC) degeneration occurs in blinding diseases such as glaucoma and other optic neuropathies. Ca^2+^ function in RGCs has been primarily studied regarding its association with neuronal activity. However, the role of RGC Ca^2+^ homeostasis and its dysregulation following degeneration is less clear. Here, we describe recent findings using rodent models of RGC degeneration that suggest a role for Ca^2+^ homeostasis in RGC loss and neuroprotection.

**Rodent models of retinal ganglion cell degeneration:** Two acute murine models of optic neuropathy are widely used to study RGC degeneration: optic nerve crush (ONC), and glaucoma models, which use different strategies to induce elevated intraocular pressure (IOP). ONC leads to injury of all RGC axons and about 20% survival by two weeks post injury (Tran et al., 2019; Guo et al., 2021; McCracken et al., 2023). Chronic IOP models of glaucoma using laser ablation, microbeads, or silicone oil to perturb anterior chamber outflow are somewhat variable in severity, leading to partial RGC axon injury and between 15%–30% RGC survival at 4–8 weeks post-induction (Guo et al., 2021; Li et al., 2022; Quintero et al., 2022). Intracellular Ca^2+^ dysregulation is thought to occur within RGCs in glaucoma (Li et al., 2022; Quintero et al., 2022), but it is unclear exactly if and how intracellular Ca^2+^ homeostasis is altered prior to RGC death. Here, we briefly discuss the current understanding of the role of Ca^2+^ in RGC degeneration.

**Ca**^**2+**^ **as an acute signal in retinal ganglion cell axon injury:** RGCs are projection neurons that have axons traversing from the eye to multiple brain regions. Their axons are particularly vulnerable at the lamina cribrosa, a specialized structure positioned at the transition from eye to optic nerve that is defective in most glaucoma patients. RGCs are thought to die in glaucoma due to retrograde signals triggered by axon injury. Axon trauma ruptures the plasma membrane, exposing intracellular contents to extracellular milieu and Ca^2+^ quickly flows down its concentration gradient into the axon. A large Ca^2+^ influx has been observed *in vivo* in rat optic nerve axons that lasted for approximately 40 seconds after optic nerve ligation and normalized within the next minute (Knoferle et al., 2010). Additionally, single axon laser ablation of cultured mammalian peripheral neurons found that injury-induced axon Ca^2+^ transients back propagate from the site of injury to neuronal somata (Cho et al., 2013), which activated regenerative cellular programs. However, regenerative Ca^2+^-mediated signaling cascades were not stimulated in RGCs following ONC (Cho et al., 2013). To determine if RGCs sense Ca^2+^ influx in their somas, we examined Ca^2+^ injury signals in RGCs with *in vivo* Ca^2+^ imaging of RGC cell bodies and their retinal axon fascicles. (Wang et al., 2021). ONC, which damages RGC axons 200–500 μm behind the optic nerve head, did not result in observable Ca^2+^ elevations in RGC axons or somas within the retina proper (McCracken et al., 2023). However, laser burn lesions in the retina proper demonstrated that RGC axon injury can lead to local Ca^2+^ elevations, but that they only propagate 200–300 μm along the axon (McCracken et al., 2023). Together, these data suggest that while Ca^2+^ can propagate over short distances in RGC axons, long-distance Ca^2+^ signaling is not relevant in RGC responses to axon injury since Ca^2+^ does not back-propagate from the optic nerve to RGCs within the retina. However, IOP elevations present in glaucoma may represent an alternative source of Ca^2+^ dysregulation.

**Assessing Ca**^**2+**^ **in chronic retinal ganglion cell degeneration using longitudinal imaging:** Longitudinal Ca^2+^ imaging of degenerating RGCs was first performed in rats with confocal laser scanning of the Ca^2+^ sensor Oregon-Green BAPTA-dextran retrogradely labeling RGCs by injection into the superior colliculus (Prilloff et al., 2007). In both partial ONC and full nerve transection, RGCs that died by 6 days after injury demonstrated strong Ca^2+^ elevations beginning 2 days after injury, suggesting that Ca^2+^ dysregulation resulting in cytoplasmic elevation drives RGC death. More recently, genetically encoded FRET-based ratiometric Ca^2+^ biosensors have been applied to RGC Ca^2+^ dynamics after ONC in mice, providing a more accurate readout of absolute Ca^2+^ concentrations. In contrast, this approach failed to find a relationship between Ca^2+^ dynamics and RGC survival after ONC (McCracken et al., 2023). Specifically, Ca^2+^ levels were relatively stable both acutely and chronically after ONC in RGC axons and somas, even at the time points just prior to cell death. The conflicting results between these two studies could be explained by a difference in animal models (rat *vs*. mice), light source for Ca^2+^ imaging, and thus background activation state (1-photon confocal *vs*. 2-photon), or the biosensor (intensity *vs*. ratiometric). As the extent of homeostatic Ca^2+^ dysregulation is unclear in ONC and unknown in IOP-induced glaucoma models, further longitudinal imaging studies investigating the role of homeostatic Ca^2+^ and intracellular mechanisms of Ca^2+^ regulation in RGC degeneration are warranted.

**Influences and alterations of Ca**^**2+**^ **handling in retinal ganglion cell degeneration:** Intracellular Ca^2+^ has been primarily studied through its association with neuronal activity, where Ca^2+^ levels rapidly increase as neurons fire action potentials. Changes in neuronal activity that occur during RGC degeneration have been investigated *in vivo* using Ca^2+^ sensors or implantable electrode arrays. In rat partial ONC models, imaging Oregon Green-BAPTA responses to light flashes showed that RGCs were less responsive to stimulation after ONC (Prilloff et al., 2007). Similarly, implanted microelectrode array recordings in mice after ONC found that RGC degeneration was preceded by reductions in spontaneous and light-driven activity. Importantly, resilient RGCs were more likely to retain their firing rates after ONC, and sustained RGCs that fire more were more likely to be resilient (Tran et al., 2019). Indeed, independent of *in vivo* measurement, RGC types that are well-characterized as having high spontaneous and/or activity-driven firing rates (e.g., alpha-RGCs and intrinsically photosensitive RGCs) are well-documented as being resistant to degeneration (Li et al., 2016; Tran et al., 2019; McCracken et al., 2023).

High neuronal activity is a feature of some resilient RGCs and can lead to Ca^2+^ elevations, but they are generally transient and persist over millisecond to hundreds of millisecond timescales. However, homeostatic Ca^2+^ levels, in which Ca^2+^ concentrations are measured over tens of seconds to minutes long time scales, are also diverse across RGCs (McCracken et al., 2023). Two-photon recordings of homeostatic Ca^2+^ levels that were not completely associated with RGC activity observed that cells with higher Ca^2+^ levels are more resilient to degeneration induced by ONC, both within and across RGC types (McCracken et al., 2023). Importantly, reducing Ca^2+^ levels using the Ca^2+^ chelator EGTA-AM reduced RGC survival, particularly in RGCs that demonstrated higher homeostatic Ca^2+^ levels. In this study, only a portion of the homeostatic Ca^2+^ levels were accounted for by neuronal activity (R activity *vs.* Ca^2+^ = 0.32), thus, determining what other cellular factors drive differences in homeostatic Ca^2+^ levels could elucidate the somewhat stochastic nature of RGC survival in degeneration.

Ca^2+^ levels related to RGC activity have been less examined in models of glaucoma. A recent study observed light-induced Ca^2+^ responses longitudinally after both ONC and glaucoma using confocal scanning laser ophthalmoscopy of the genetically encoded Ca^2+^ sensor jGCaMP7s (Li et al., 2022). This paradigm classified RGCs into ON, OFF, and ON-OFF dynamic response types after large flashes of full-field ultraviolet light. Surprisingly, RGCs were found to switch response characteristics in both degeneration models. Comparing across models further showed that Ca^2+^ dynamics were altered differently in the different forms of degeneration. ON-RGC responses were more common 1 and 3 days after ONC, but by 5–14 days post ONC, they were almost completely gone. OFF-RGC responses decreased gradually. However, in a silicone oil glaucoma model ON-RGCs retained their responses, and OFF-RGCs converted to ON-RGCs. The only phenomena consistent with both ONC and IOP glaucoma was that OFF-Transient and OFF-Sustained RGCs were the most susceptible (Li et al., 2022). To the best of our knowledge, this is the only study assessing Ca^2+^ in both ONC- and IOP-induced glaucoma models of RGC degeneration with longitudinal *in vivo* imaging. In separate experiments using acute transscleral 2-photon imaging of albino mice expressing GCamp in RGCs in a microbead glaucoma model, Ca^2+^ dependent spike amplitudes responding to laser scanning onset were decreased, and latencies were increased (Quintero et al., 2022). Taken together, intrinsic and circuit-influenced changes in Ca^2+^ dynamics likely occur over the course of RGC degeneration. These results indicate different changes in activity-dependent Ca^2+^ during neurodegeneration, highlighting the need to study mechanisms of RGC loss in IOP-based glaucoma models along with ONC.

**Figure 1:**
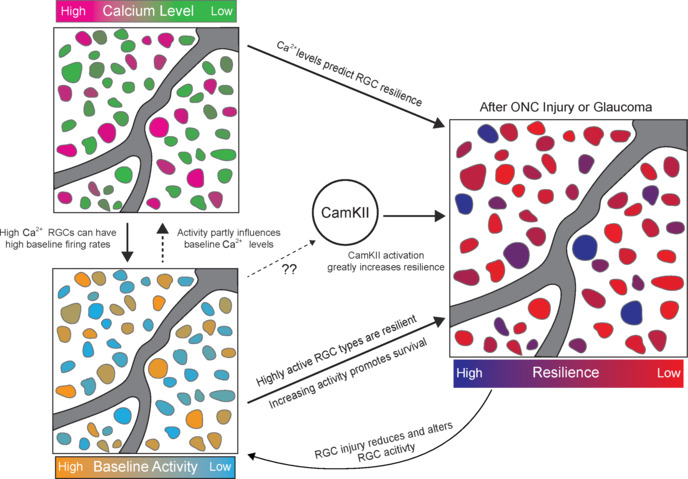
Relationship between Ca^2+^, circuit activity, and RGC resilience to degeneration. CamKII: Calcium–calmodulin dependent protein kinase II; ONC: optic nerve crush; RGC: retinal ganglion cell.

**Modulating Ca**^**2+**^ **signaling and circuit activity for retinal ganglion cell neuroprotection and regeneration:** Ca^2+^ signaling pathways also show promise as a treatment to boost RGC neuroprotection, where native high levels of intracellular Ca^2+^ and circuit activity are a trait of RGCs that resist degeneration (**Figure 1**). Furthermore, increasing RGC activity promotes axon regeneration and survival. Observed decreases in RGC activity after ONC axon injury have been partially attributed to the hyper-activation of amacrine cells that leads to hyper-inhibition of RGCs. When this amacrine cell inhibition is reduced with either gene therapy or pharmacological interventions, RGC survival is increased and RGCs regenerate in response to growth factors (Zhang et al., 2019). Directly stimulating RGC activity with activating DREADDs also induces increased RGC axon regeneration and survival (Li et al., 2016). Similarly, increasing RGC activity via visual stimulation enhances RGC survival and regeneration when combined with pro-regenerative treatments targeting the mTOR pathway (Lim et al., 2016).

The intracellular pathways downstream of Ca^2+^ that may be critical for RGC resilience to injury remain unclear. However, CaMKII signaling acts as a strong neuroprotective signaling hub in both ONC and glaucoma models. Overexpression of constitutively active CaMKII components leads to near complete survival of RGCs in mouse models of glaucoma and ONC through CREB-mediated transcription (Guo et al., 2021). Interestingly, boosting CaMKII signaling did not promote axon outgrowth, despite its strong neuroprotective effects, suggesting that activity-mediated axon regeneration involves additional signaling components and that constitutively activating CAMKII is independent of neuronal activity. Examination of natively resilient RGCs with high homeostatic Ca^2+^ levels found no evidence of higher CaMKII or CREB signaling either at baseline or after ONC as compared to low Ca^2+^ RGCs (McCracken et al., 2023), indicating that higher homeostatic Ca^2+^ set points are not directly associated with CAMKII signaling. This lack of association suggests that other Ca^2+^ signaling pathways related to homeostatic Ca^2+^ could be neuroprotective and distinct from CAMKII signaling.

In summary, RGCs demonstrate a somewhat unexpected relationship between Ca^2+^ and cell resiliency in that higher homeostatic Ca^2+^ levels and dynamics are traits of RGCs that persist in neurodegenerative conditions. Degenerative conditions including ONC and glaucoma reduce RGC activity and likely Ca^2+^. Although intracellular Ca^2+^ homeostasis is recognized as crucial for cellular processes, its role as a primary mechanism in RGC survival remains uncertain, particularly in glaucomatous degeneration. It is worth noting that excitotoxicity leads to a cascade of Ca^2+^ influx that induces neuronal death in many neurodegenerative conditions. This perspective highlights the lack of a negative role for Ca^2+^ influx in RGCs after ONC. Rather, it is emphasized that an increase in Ca^2+^ homeostasis and neuronal activity is beneficial for RGCs, an idea that is contrary to the excitotoxic model of neuronal death. High activity demands and mechanisms for elevated Ca^2+^ homeostasis are likely below thresholds for Ca^2+^ related excitotoxicity. More in-depth research to determine whether disruptions in Ca^2+^ balance directly contribute to RGC death or if these changes are secondary to other degenerative processes associated with glaucoma is necessary. Understanding this relationship is vital for developing effective treatments for glaucoma and related conditions.

**Additional file:** *Open peer review report 1.*

OPEN PEER REVIEW REPORT 1
