# Supplementary material for: Role of calcium homeostasis in retinal ganglion cell degeneration
Source: Neural Regen Res. 2025 Apr 29;21(5):2009–10. doi: 10.4103/NRR.NRR-D-24-01651 (PMC12694647; doi:10.4103/NRR.NRR-D-24-01651)
Supplement: Supplementary file 1 [file NRR-21-2009_Suppl1.pdf]

OPEN PEER REVIEW REPORT 1

Name of journal: Neural Regeneration Research
Manuscript NO: NRR-D-24-01651
Title: Role of calcium homeostasis in retinal ganglion cell degeneration
Reviewer's Name: Simone Ebert
Reviewer's country: France

COMMENTS TO AUTHORS

The manuscript is overall well-written, structured and interesting.
The short perspective covers known findings about changes in calcium homeostasis and activity dependent calcium levels in degeneration and neuroprotection of retinal ganglion cells. It briefly covers main models of mechanic RGC degeneration, then highlights known findings about acute and longitudinal changes in CA2+ levels as well as activity induced changes in Ca2+ levels in neurodegeneration and neuroprotection.
It discusses that intracellular calcium levels acutely increase after axonal injury, while longitudinal changes in calcium levels remain largely unclear. The effects of these changes in calcium homeostasis on degeneration are unclear. The main findings highlighted are that high Ca2+ baseline levels and high neural activity promote neural resilience and that increases of CamKII activity, a downstream factor in the Ca2+ signalling cascade, can increase cell survival. It overall emphasizes the need for further investigation in these directions.

The reader has several minor comments:

Section 2:
- Lines 19-24, the statment that Ca2+ transients backpropagate to the some contacdicts the the rest of the paragraph. This should be clarified.
- Lines 26-34 on p.2, are these new, unpublished results?

Section 3 and 4:
- Distintction between activity dependent Ca2 levels and homeostatic Ca2 levels seem key to determine roles of Ca2+ in degeneration and regeneration, but is somewhat difficult to entangle in sections 3 and 4 of the paper. The only paragraph about homeostatic levels seems to be in lines 21-39 on p.4.
- Paragraph lines 14-39 on p.5 talks about downstream signalling effects of Ca2+. Is the observed effect of CaMKII due to its involvement of calcium homeosthatis or a consequence of increased intracellular calcium ? In this study, was CaMKII activity related to neuronal activity?

- Section 3 (degeneration) already talks about the role of neural activity in neuroprotection, which seems slightly redundant is the scope of such a short article.

Overall, the beneficial effect of increased activity and calcium levels is somewhat surprising, given that excitotoxicity seem to be a major driver for many other neurodegenerative diseases. This could be commented.
